# Frailty and Mortality in a Community-Dwelling Relatively Healthy Older Population

**DOI:** 10.1093/geroni/igab046.2192

**Published:** 2021-12-17

**Authors:** A R M Saifuddin Ekram, Joanne Ryan, Sara Espinoza, Michael Ernst, Anne Murray, Robyn Woods

**Affiliations:** 1 Monash University, Monash University, Melbourne, Victoria, Australia; 2 Monash University, Melbourne, Victoria, Australia; 3 University of Texas Health Science Center San Antonio, San Antonio, Texas, United States; 4 University of Iowa, Iowa City, Iowa, United States; 5 Hennepin HealthCare Research Institute, Hennepin HealthCare, Minneapolis, Minnesota, United States

## Abstract

This study examined factors associated with frailty and studied the association between frailty status and mortality in healthy community-dwelling older persons. Participants included 19,114 individuals from the “ASPirin in Reducing Events in the Elderly” (ASPREE) trial. Frailty was defined using modified Fried phenotype comprising exhaustion, body mass index, grip strength, gait speed and physical activity. A deficit accumulation frailty index (FI) using 66 items was also developed. Correlates of frailty were examined using multinomial logistic regression. The association between frailty status at baseline and mortality was analyzed using Cox regression. At baseline, 39.0% (95% CI: 38.3, 39.7) of participants were prefrail, and 2.2% (95% CI: 2.0, 2.4) were frail according to Fried phenotype, while 40.6% (95% CI: 40.0, 41.3) of participants were pre-frail and 8.1% (95% CI: 7.7, 8.5) were frail according to FI. Older age, female sex, lower education, African-American and Hispanic ethno-racial status, smoking, alcohol use, comorbidities, and polypharmacy were associated with frailty status. Pre-frailty increased risk of all-cause mortality significantly (Fried HR: 1.48; 95% CI: 1.28, 1.71; FI HR: 1.54; 95% CI: 1.31, 1.81); and the risk was even higher for frailty (Fried HR: 2.24; 95% CI: 1.67, 3.00; FI HR: 2.34; 95% CI: 1.83, 2.99) after adjustment for covariates. Cardiovascular disease (CVD) and non-CVD-related mortality showed similar trends. These results highlight a considerable burden of pre-frailty among a large group of community-dwelling, initially healthy older adults. Both Fried phenotype and deficit accumulation FI similarly predicted all-cause, CVD and non-CVD-related mortality in relatively healthy older adults.

